# A Cross-Sectional Comparison of Perceived Quality of Primary Care by Hypertensive Patients in Shanghai and Shenzhen, China

**DOI:** 10.1097/MD.0000000000001388

**Published:** 2015-08-28

**Authors:** Haitao Li, Xiaolin Wei, Martin Chi-Sang Wong, Samuel Yeung-Shan Wong, Nan Yang, Sian M. Griffiths

**Affiliations:** From the School of Medicine, Shenzhen University, Shenzhen, China (HL) and School of Public Health and Primary Care, Faculty of Medicine, The Chinese University of Hong Kong, Hong Kong, China (XW, MC-SW, SY-SW, NY, SMG)

## Abstract

Hypertension should be best managed under primary care settings. This study aimed to compare, between Shanghai and Shenzhen, the perceived quality of primary care in terms of accessibility, continuity, co-ordination, and comprehensiveness among hypertensive patients.

A cross-sectional study was conducted in Shanghai and Shenzhen, China. Multistage random sampling method was used to select 8 community health centers. Data from primary care users were collected through on-site face-to-face interviews using the primary care assessment tool. Good quality standard was set as a value of 3 for each attribute and a value of 18 for total score.

We included 568 patients in Shanghai and 128 patients in Shenzhen. Compared with those in Shenzhen, hypertensive patients in Shanghai reported a higher score in co-ordination of information (3.37 vs 3.66; *P* < 0.001), but lower scores in continuity of care (3.36 vs 3.27; *P* < 0.001), and comprehensiveness-service provision (3.26 vs 2.79; *P* < 0.001). There was no statistically significant difference in total scores between the 2 cities (18.19 vs 18.15). Over 3-quarters of hypertensive patients in both cities reported accessibility (97.2% vs 91.4%) and co-ordination of services (76.1% vs 80.5%) under good quality standard, while <1-quarter of them rated continuity of care (23.6% vs 22.7%), co-ordination of information (4.8% vs 21.1%), and comprehensiveness-service availability (15.1% vs 25.0%) under that standard.

Compared with Shenzhen, the perceived quality of primary care for hypertensive patients in Shanghai was better in terms of co-ordination of information, but poorer on continuity of care and comprehensiveness-service provision. Our study suggests that there is room for quality improvement in both cities.

## INTRODUCTION

International experience has shown that strong primary care is associated with better health outcomes, lower costs, and better distribution of health across and within populations.^1^ Primary care is thus widely recognized by policy makers to be the corner stone of any healthcare system. The World Health Organization (WHO) report of 2008 urged WHO member states to strengthen their health systems through the principles and values of primary care.^2^ In 2009, the Chinese Government launched the New Healthcare Reform Plan, placing great emphasis on primary care for “Health for All,” and strong primary care is urgently needed.^3^ However, much remains unknown regarding how to make the necessary improvements. Quality assessment is part of ensuring effective delivery of primary care, and maximizing its full potential. Primary care quality can be measured by accessibility, continuity, co-ordination, and comprehensiveness, which are recognized as the key attributes of a primary care process.^4,5^ The internationally recognized primary care assessment tool (PCAT) is widely used to measure these attributes from the patients’ viewpoint.^6–11^

Hypertension is an important public health issue faced by worldwide policymakers including China. According to the World Health Statistics 2012 report,^12^ 1 in 3 adults had elevated blood pressure (BP) in the world. It is estimated that, in 2025, there will be 1.56 billion adults living with hypertension.^13^ The prevalence of hypertension in the Chinese population has increased sharply during the past several decades, and has reached a rate of 34% among adults aged 25 years and above in 2010.^14^ Hypertension is recognized by the WHO as one of the most important causes of premature death. It is estimated that hypertension causes about 7.5 million deaths annually, accounting for 12.8% of total deaths in the world.^15^ In the Chinese population, it is estimated that about 50% of deaths are attributable to prehypertension and hypertension.^16^ In response, the Chinese Government designs chronic disease management as 1 of the 6 integrated health services, provided by primary care organizations.^17^ The management and control of hypertension have become priorities at primary care organizations, and hypertension is an important focus of primary care. However, currently <20% of hypertensive patients had their BPs optimally controlled.^18,19^

The primary care system is the foundation of the 3-tier health system in China. It is commonly consists of community health centers (CHCs) in urban areas and township hospitals in rural areas. CHCs were firstly established by government or public hospitals in 1997. During the past decade, there has been a rapid expansion in the number of CHCs. In 2009, the proportion of cities with CHCs offering primary care to the public reached over 90%.^20^ Primary care providers in CHCs usually include physicians, nurses, and public health practitioners. Six-integrated health services are designed to be provided by CHCs including medical care, preventive care, rehabilitation, chronic disease management, health education, and promotion and technical support for family planning. Government funding, health insurance reimbursement, and out-of-pocket payments are 3 major sources of revenues of CHCs. CHCs in China are walk-in clinics, while patients may seek healthcare directly from secondary or tertiary hospitals without being referred by primary care providers.^21^ Due to the socioeconomic variations in different urban regions, 2 major models of CHCs have emerged: government-managed CHCs (G-CHCs) and hospital-managed CHCs (H-CHCs), together accounting for about 86% of all CHCs.^22^ Shanghai and Shenzhen are 2 metropolitan cities in China with comparable economic levels, but which have developed different CHC models. CHCs in Shanghai are G-CHCs, directly managed and fully funded by the government, and independent of public hospitals.^23^ Shenzhen's CHCs are H-CHCs that are directly managed by public hospitals as a department. Local government plays a more important financial and supervisory role among CHCs in Shanghai than in Shenzhen, while hosting hospitals play more important financial and administrative roles among CHCs in Shenzhen. Although, the CHCs in both cities are publicly owned, the CHCs in Shenzhen rely heavily on revenue-generating activities for financial survival. The number of CHC health workers in Shanghai is usually larger than that in Shenzhen.

A number of international studies have been conducted to investigate the quality of primary care for hypertensive patients. The studies are either from a health professional's perspective or from a patient's perspective, which together forms the 2 basic perspectives held by stakeholders concerning quality of hypertensive care.^24^ The indicators used range from initial screening, through diagnosis, treatment and follow-up to the outcome, which are from a health professional's perspective.^25–30^ Patient's satisfaction is an indicator usually used from a patient's perspective.^31–33^ In China, most of the current studies, investigating quality of hypertensive care, used rates of hypertension control or drug prescriptions or patient's satisfaction as quality indicators.^19,34,35^ To the best of our knowledge, few have studied on the perceived process quality of primary care among hypertensive patients. The different ownership types of CHCs determine to whom and for what they are held accountable, which would consequently influence the delivery of hypertensive care. Although previous studies showed mixed views regarding the quality of hypertensive care delivered by different models of primary care facilities, a quality comparison between 2 models of CHCs in the 2 cities can help identify defects among hypertensive patients for quality improvement. This study aimed to compare hypertensive patients’ perceptions of the quality of primary care in Shanghai and Shenzhen, China, in terms of accessibility, continuity, co-ordination, and comprehensiveness. As both cities are leading primary care development in China, our study may help identify gaps in hypertensive care delivery in the 2 cities and provide valuable feedbacks that other cities can use as benchmarks to assess their own quality of primary care for hypertensive patients.

## METHODS

### Ethical Approval

Ethical approval was obtained from the Joint Chinese University of Hong Kong and New Territories East Cluster Clinical Research Ethics Committee (Ref. No. CRE-2010.441).

### Study Design and Settings

A cross-sectional study was conducted in Shanghai (in November 2011) and Shenzhen (in June 2012), China. Employing a multi-stage stratified random sampling method, CHCs were chosen as study settings. In the first stage in Shenzhen, 10 districts were stratified into 4 geographical areas within or outside Shenzhen Economic Zone, and in the eastern or western part of the city. In Shanghai, the 16 districts were divided into 4 geographical areas in the eastern, western, southern, or northern part of the city. By using simple random sampling methods, we randomly selected 1 district in each category to arrive at 4 districts in each city; these included Jing’an, Changning, Xuhui, and Pudong in Shanghai, and Futian, Luohu, Bao’an, and Longgang in Shenzhen. In the second stage, we selected 1 CHC in each district in Shanghai using simple random sampling methods. In Shenzhen, using simple random sampling methods, 1 hosting hospital was firstly chosen from each randomly drawn district; 1 CHC was then randomly selected from each recruited hosting hospital. In total, 8 CHCs were selected as study settings.

### Sampling Frame and Procedures

With a mean difference of 1.2 and standard deviation of 3.4 for total PCAT score among hypertensive patients,^7^ we estimated that a minimum sample size of 126 in each city was needed to generate a 95% confidence level and 80% statistical power. This study was part of the project funded by the RGC of Hong Kong which intended to assess primary care development in Shanghai, Shenzhen, Kunming, and Hong Kong. In that project, the sampling frame was CHC users’ population based. Patient inclusion criteria were: age of 18 years and above; ability to communicate and give informed consent; and at least 1 CHC visit before survey. Using a systematic sampling design, every 5th care user was selected until at least 200 participants in each CHC had been recruited. Extensively trained interviewers performed face-to-face surveys. Participants were assured of the anonymity and confidentiality of the survey, and informed consent was obtained before the surveys commenced. In total, 811 participants in Shanghai and 806 in Shenzhen completed the survey with the response rates of 94% and 85%, respectively. Of those, 568 from Shanghai and 128 from Shenzhen were patients with hypertension and were included in this study.

### Key Measures

The adapted and validated Chinese version of the PCAT was used to collect data.^36^ The questionnaire asked about primary care attributes, including accessibility, continuity, co-ordination of services and information, comprehensiveness-service availability and provision, which were used as indicators to monitor primary care systems. Accessibility referred to the ease with which a patient could obtain needed care for any health problem with primary care provider, indicating organizational or structural accessibility. Continuity of care meant the care over time by a single primary care provider and the nature of the relationship. Co-ordination included some form of informational continuity, as well as integration of problems addressed elsewhere into the total care of the patients. Comprehensiveness measured the range of all types of health services delivered by CHCs and the actual receipt of indicated specific services by the patients. Each question was scored on a 4-point Likert-type scale, with higher scores indicating higher quality. Performance relating to each attribute was presented as a mean score for all questions under the subject heading (range, 1–4). A total score, referring to overall primary care quality, was created by adding the scores for each attribute (range, 6–24). Additionally, good quality standard, which represented a quantitative expression of patient's expectations of primary care, was set as a value of 3 for each attribute and a value of 18 for total score.^37^ We defined that the percentages of those who reported a score under the quality standard ≥75% (≤25%) as poor (good) quality of care.^38^

Socio-demographic characteristics and healthcare measures were also collected, including gender, age, marital status, household register, education level, occupation status, household income, health insurance, health status, number of CHC visits, and length of time with the CHC. The distribution of categorical and continuous variables was evaluated, and appropriate cut-points were determined by the distribution of variables and also based on prior literature. For marital status, we classified the participants into 2 groups: those single (including not married, widowed, and divorced) and those currently married. According to household register, respondents were grouped into local residents and migrants. Migrants were internal migrants, which refer to those who do not change their official *Hukou* registration to the new location which they move to.^39^ Education level was collapsed into 3 categories: less than middle school, high school diploma and equivalent, and college and above. We simply classified occupation status into 2 groups: those who have a job (including employed and self-employed) and those who do not have a job (including the unemployed, retired, and housewives). The participants were classified into 3 economic groups depending on monthly household poverty line (RMB3000/US$484) and mean household income level (RMB10,000/US$1282) in 2011.^40,41^ Information on health insurance was also collected. Participants with health insurance were those covered by any type of social health insurance scheme. In Shenzhen, health insurance scheme included Medical Insurance Scheme for Migrant Employees and Comprehensive Health Insurance Scheme, while in Shanghai, health insurance scheme consisted of Basic Medical Insurance Scheme for Urban Employees and Basic Medical Insurance Scheme for Urban Residents. To understand the degree of the participants’ familiarity with CHCs, we asked the number of CHC visits during the past 1 year period and their length of time with the CHC since their first visit.

### Statistical Analysis

We compared the participants’ socio-demographic characteristics and healthcare measures between the 2 cities using Chi-squared tests. The differences in mean scores of selected items, individual and total primary care attributes between the 2 cities, were firstly examined by independent 2-sample tests. Next, multiple linear regression models were constructed to compare between the 2 cities after controlling for all participants’ socio-demographic characteristics (sex, age, marital status, household register, educational level, employment, household income, health insurance, and health status), and healthcare measures (CHC visits and length of time with CHC). Model fittings were conducted using backward elimination with a threshold of 0.10 for variable inclusion in the model. Results were presented as β with 95% confidence interval (95% CI). The percentages of those who reported a score below the good quality standard in all individual and total primary care attributes were presented. For all tests conducted in the study, a *P* value of <0.05 was adopted as the statistically significant level. All analyses were conducted using SPSS 19.0.

## RESULTS

Participants differed, between the 2 cities, in all socio-demographic characteristics and healthcare measures except for educational level. More than half of the participants in both cities were female, while those in Shanghai (68.8%) were more likely to be female compared with those in Shenzhen (53.9%). Majority of the participants in Shanghai and Shenzhen were married (86.3% vs 95.3%) and aged more than 60 years old (77.5% vs 65.6%). The participants in Shenzhen tended to be migrants (68.8%), while the participants in Shanghai tended to be locals (96.3%). About half of the participants in both Shanghai and Shenzhen had an education of middle school or below level (45.8% vs 46.9%); 92.4% of the participants from Shanghai described themselves as retired, housewives, or unemployed, while that figure was only 62.5% for Shenzhen. Compared with the counterparts in Shanghai, the participants in Shenzhen were more likely to have high incomes (8.5% vs 31.3%), while about 1/3 refused to report their income in Shenzhen. The majority of the participants in Shanghai were insured by at least 1 type of health insurance scheme, which was 41.1% higher than that in Shenzhen (57.8%). More than 2/3 of the participants in both cities reported fair or poor health status, 83.3% and 71.9%, respectively. The reported number of visits to CHCs by the participants in Shanghai was greater than that in Shenzhen (*P* < 0.001). The participants from Shanghai had been utilizing services provided by the CHCs for a longer period than their counterparts from Shenzhen (*P* = 0.001) (Table 1).

**TABLE 1:**
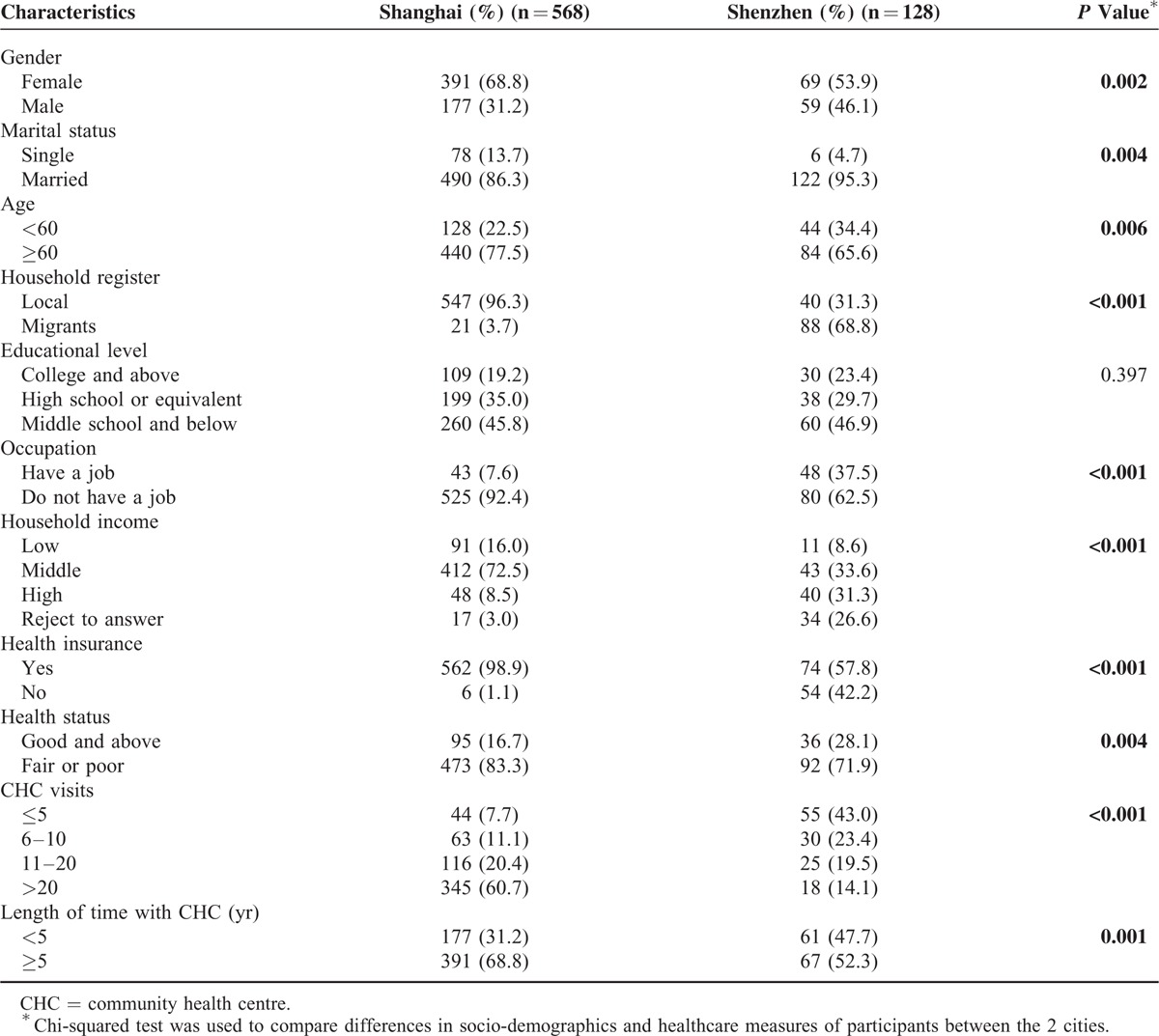
Socio-Demographic Characteristics and Healthcare Measures of the Participants by City

After controlling for confounders, it was found that participants in Shenzhen reported higher scores in continuity of care (3.27 vs 3.36; *P* < 0.001) and comprehensiveness-service provision (2.79 vs 3.26; *P* < 0.001) when compared with those in Shanghai. However, Shanghai participants reported a higher score in co-ordination of information than those in Shenzhen (3.66 vs 3.37; *P* < 0.001). There was no significant difference in total score (18.15 vs 18.19) between the 2 cities (Table 2).

**TABLE 2:**
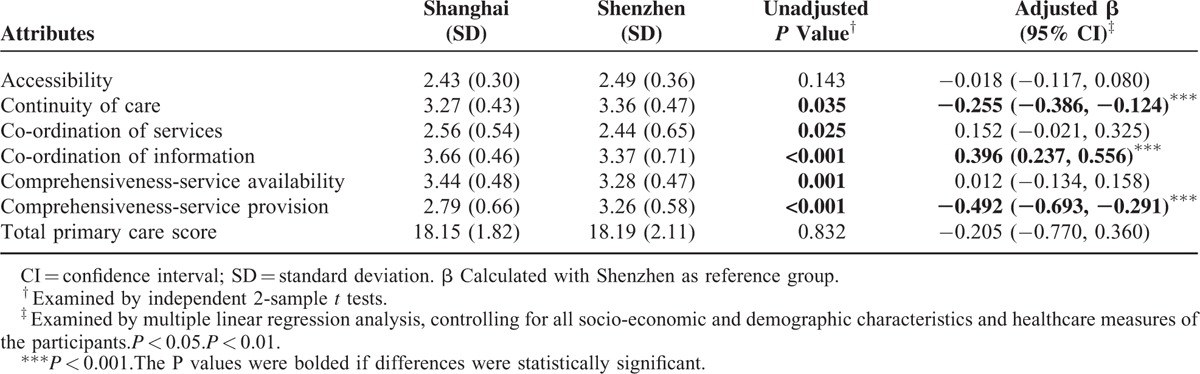
Individual and Total Primary Care Attributes Scores Reported by the Participants by City

Further analyses were performed to compare the selected items between the 2 cities after adjusting for confounders (Table 3). Regarding accessibility, participants in both cities tended to wait more than 30 min in CHCs before receiving needed care, scoring 2.41 in Shanghai and 1.97 in Shenzhen. While more Shenzhen participants perceived that CHCs were open at least until 8 pm on some weekday evenings (2.25 vs 3.65; *P* < 0.001), the Shanghai participants nevertheless reported greater ease of obtaining care from CHCs (3.21 vs 1.35; *P* < 0.001). As for continuity of care, more participants in Shenzhen than in Shanghai believed that their doctors understood what they said or asked (3.81 vs 3.84; *P* < 0.05) and their questions were answered in ways they understood (3.81 vs 3.86; *P* < 0.001). Compared with those in Shanghai, participants in Shenzhen felt their doctors were more likely to know their most important health problems (*P* < 0.05), complete medical history (*P* < 0.05), and employment status (*P* < 0.001).

**TABLE 3:**
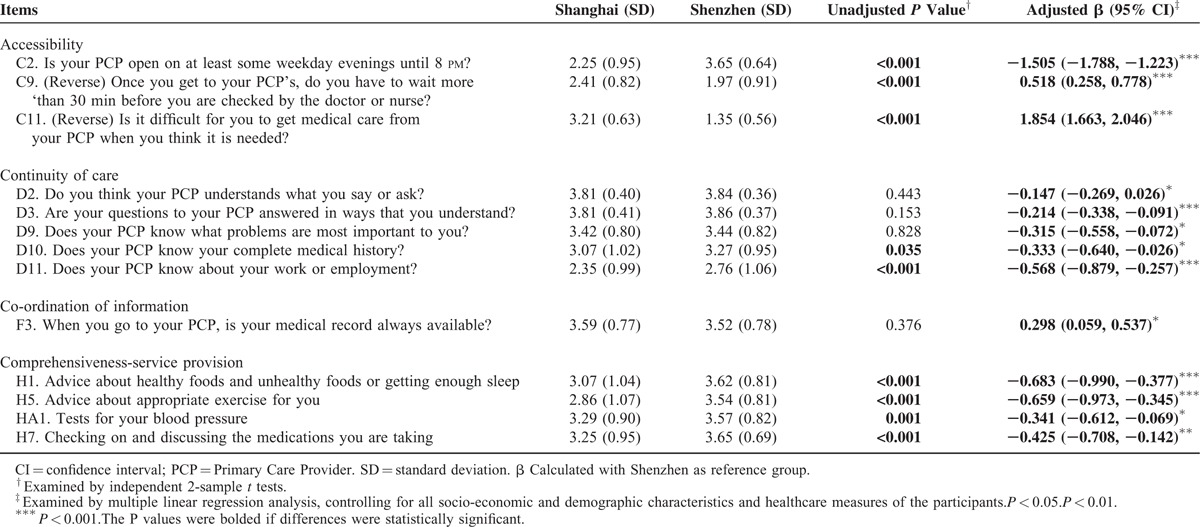
Reported Mean Scores of the Selected Items Under the Selected Attributes by City

Over 3-quarters of the participants in Shanghai and Shenzhen reported the scores of access to (97.2% vs 91.4%) and coordination of services (76.1% vs 80.5%) below the benchmark of 3. However, less than 1-quarter of the participants rated below the benchmark on such attributes of primary care as continuity of care (23.6% vs 22.7%), co-ordination of information (4.8% vs 21.1%), and comprehensiveness-services availability (15.1% vs 25.0%) (Table 4).

**TABLE 4:**
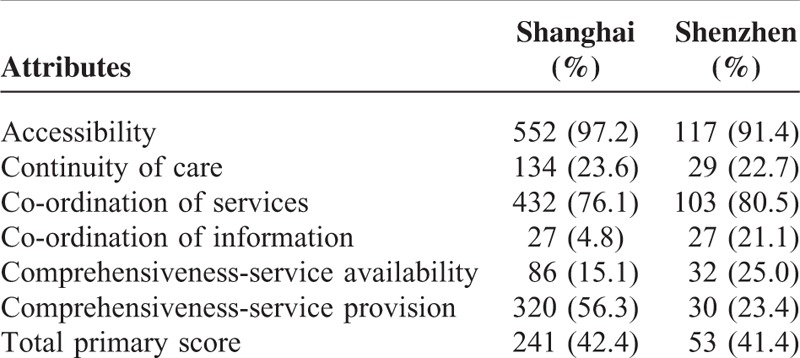
Percentages of the Rating Scores Under Good Quality Standard Level by City

## DISCUSSION

Our study showed that compared with those in Shenzhen, the hypertensive patients in Shanghai experienced better co-ordination of information, but poorer continuity of care and comprehensiveness-service provision. The hypertensive patients in both cities had poor experiences in accessibility and co-ordination of services, but good experiences in continuity of care, co-ordination of information, and comprehensiveness-service availability. Between respondents of the 2 cities, no significant difference was identified in overall primary care quality.

The limitations of the study should be considered. Firstly, caution should be made to the general applicability of the findings at the city level since only 4 CHCs were selected in each city. Besides, conclusions were based on information supplied by CHC users and cannot be extended to the population in general. Secondly, the representativeness of the study is limited. Only 4 CHCs were selected in each as study settings which might introduce sampling bias, although we used stratified random sampling method to select CHCs. Moreover, due to the nonavailability of the exact sampling frame of patients seeking health services from the sampled CHCs on a certain day, we did not use random sampling methods to select the participants; however, the systematic sampling method replicated the random sampling method effectively. Furthermore, selection bias might be introduced since the sampling frame is not specifically designed for the present study. Thirdly, given that the scores were patient-reported, our estimates might be subject to recall bias not accounted for by statistical adjustments; however, there is no reason why the recall bias would occur systematically. Moreover, the on-site face-to-face interview survey approach could minimize this recall bias. Fourthly, since the differences in socio-demographic characteristic between the participated and refused participants were unknown, nonresponse bias might be introduced although the response rates were high in both cities. Finally, clinical indicators of hypertensive patients were not collected in our study. Patients encountered in CHCs from the 2 cities might have different severity of hypertension and length since hypertension diagnosis, etc.; they thus might have different experiences of care that might ultimately bias the results.

Hypertensive patients in Shenzhen reported a higher score in continuity-of-care than those in Shanghai, which indicated a better relationship between hypertensive patients and primary care providers in Shenzhen than that in Shanghai. The hypertensive patients’ perceptions of understanding might result in increased levels of confidence and trust in their doctors and in the care they receive, and consequently improve continuity of care.^42^ In our study, more participants and CHC doctors in Shenzhen understood each other well than in Shanghai. The differences in health insurance schemes between the 2 cities might be another possible explanation of this finding. In Shanghai, hypertensive patients can choose healthcare providers without any constraints. However, in Shenzhen, migrant workers, who comprised the majority of the participants, have to obtain referrals from their CHC doctors for re-imbursement of charges for hospital services.^43^ In other words, CHC doctors in Shenzhen act as health gatekeepers for these patients so as to know more about their complete medical history and most important health problems.

The hypertensive patients from Shenzhen also reported a higher score in comprehensiveness-service provision than those from Shanghai. This might mean that hypertensive patients from Shenzhen are more likely to receive nonpharmacological treatments to control the disease. Although there are usually more doctors in CHCs in Shanghai than in Shenzhen, the per day workload assumed by a doctor in Shanghai is heavier than that in Shenzhen, which results in the more limited time spared to each hypertensive patient in Shanghai and health education services are usually neglected. Therefore, the workload might be 1 possible explanation to the difference in service provision between the 2 cities.

However, the hypertensive patients in Shanghai reported a higher score of co-ordination of information than those in Shenzhen. This may indicate a better information-sharing system among CHCs and hospitals in Shanghai than those in Shenzhen. The utilization of electronic information systems may facilitate the keeping and referencing of medical records.^44^ In Shanghai, an information-sharing system has been established between hospitals and CHCs in each district while, in Shenzhen, only the CHCs have access to a common information system, which is separate from that of public hospitals, even their holding public hospitals. Sharing of data facilitates access to medical records among different health facilities, as seen in the Shanghai example.

Hypertensive patients in both cities had poor experience in access to- and co-ordination of services. Our study showed that hypertensive patients in both cities had to wait for more than 30 min since their arrival to CHCs to receive the needed care, which is 1 possible explanation of the poor performance in accessibility. In China, due to lack of an appointment system and an optimal scheduling system, it is not surprise that patients experienced long waiting time. Also, CHC opening times are another possible reason for hypertensive patients’ poor accessibility experience in Shanghai. Working hours of health facilities have been found as an important factor influencing the accessibility of healthcare services.^45^ PCAT questionnaire showed that the hypertensive patients in Shanghai tended to perceive that the CHCs were not open late in the evening (Table 3). While most of the hypertensive patients from the CHCs in Shenzhen perceived that it was difficult for them to get medical care when needed. In Shenzhen, because migrant employees who are not given paid leave for medical care, they may have to wait until the end of their work shifts to attend CHCs, which makes it difficult for them to get medical care.^46^ Financial incentives, regulation, and professional ethics may act as barriers to effective referral between health facilities in both cities.^47^

However, the hypertensive patients from both cities reported that they experienced good comprehensiveness-service availability. Previous studies have indicated that availability of comprehensiveness-service is associated with the original missions of primary care practices.^6,48^ The designed 6 integrated health services package guarantees the comprehensiveness of services delivered by CHCs. Multidisciplinary teamwork is another possible explanation of the good availability of comprehensiveness-service.^49^ The doctors working in CHCs consist of general practitioners and Traditional Chinese Medicine (TCM) doctors who are former specialists in different disciplines. This broad skill base ensures a wide range of treatments and services at CHCs.

Our study identified no significant difference in total score between the 2 cities, but there were significant differences in continuity of care, co-ordination of information, and comprehensiveness-service provision. The current literature showed mixed views regarding the relationship between the ownership of primary care organizations and quality of care delivered. Studies in the USA^50,51^ and Canada^52^ showed that when compared with hospital outpatient clinics, G-CHCs tended to provide a higher quality of primary care in terms of better co-ordination and comprehensiveness. A study conducted in Southern China showed that the total score reported by the participants from G-CHCs was higher than H-CHCs.^53^ However, studies in Hong Kong^44^ and South Korea^48^ indicated that perceived quality of primary care, including overall quality of care, accessibility, continuity, and comprehensiveness, offered by for-profit private or hospital outpatient clinics was better than government-owned primary care organizations. Therefore, due to lack of a clear difference in quality of care between primary care organizations with different ownership, it appears that the performance of CHCs depends more on management skills of local governments than ownership structure.^54^ However, caution should be made when the present findings were compared with international studies since the PCAT is adapted and modified according to local contexts.

It was found that there were more participants with hypertension in Shanghai than in Shenzhen, 568/811 and 128/806, respectively, which is possibly due to the different population structures between the 2 cities. In Shanghai, people aged 60 years or over account for about 14% of its total population, while in Shenzhen, the proportion is only about 3%. The prevalence of hypertension in the general population in Shanghai and Shenzhen are 31% and 14%, respectively. Moreover, the designated target populations of CHCs are the elderly and the poor, etc. Hypertension is found to be the commonest co-morbidity among the elders. Therefore, among CHC users, hypertension prevalence rate is expected to be higher in Shanghai than in Shenzhen.

Timely access to primary care can prevent hypertensive patients from becoming sicker and allow better management of the disease.^55^ Extending opening hours may be a possible means of avoiding overcrowding and shortening patients’ waiting time, and to improve their access to services. Looking at Shenzhen, new policies should be considered that encourage employers to give paid leave to their employees who are absent from work seeking healthcare. Enhanced continuity of care has been shown to be associated with better compliance with follow-up appointments,^56^ and prevents a sudden worsening in progress among hypertensive patients.^57^ To improve continuity of care, for CHCs in Shanghai, communication between patients and doctors should be strengthened: for example, the language should be simple and devoid of medical terminology for improved patient understanding. It is necessary to adopt a comprehensive approach to the control of hypertension, based on the needs and risks of different population sub-groups, to reduce the development of the disease. Despite the fact that multidisciplinary teamwork results in interruption of care from an individual doctor, the patients maintain continuity with the team under the registration system—as in the UK and Canada—which ensures quality primary care delivery.^58,59^ Building general practitioner teams, made up of general practitioners (and/or TCM doctors), public health doctors and nurses, might be a strategy for improving comprehensiveness-service availability, while not losing continuity with the team for the registered populations. On the other hand, the staffing standards of a CHC need to be updated according to the total volume of services provided by the CHC to ensure the actual provision of the integrated pharmacological and nonpharmacological treatment to hypertensive patients.^60,61^ Dual-referral system should be established between primary care providers and specialist for better management of hypertension, where co-ordinated medical records are important because they enhance doctors’ abilities to recognize hypertensive patients’ problems and therapies. The referral system might be established through regulation such as guidelines and audit, re-establishment of professional ethics and reasonable mechanisms for profit distribution among CHCs and hospitals. Furthermore, integrated information systems, shared by CHCs and hospitals, may assist with access to patients’ medical records, as with SystmOne in the UK.
